# Feasibility of DBS screening to identify adult patients with Pompe disease in a neuromuscular clinic population

**DOI:** 10.1186/1471-2474-14-S2-P1

**Published:** 2013-05-29

**Authors:** Angela Genge

**Affiliations:** 1 Montreal Neurological Institute and Hospital, Montreal, Quebec, Canada

## Introduction

In January 2012, a program was set up to rapidly screen patients with undiagnosed muscle disorders who were referred to a neuromuscular specialist in an adult hospital. A nurse was trained to do the DBS procedure as part of a series of required laboratory tests. All individuals referred to a neuromuscular specialist, either within the context of a neuromuscular clinic or an EMG laboratory, were considered. Patients with an elevated CPK, symptoms and signs indicative of a metabolic myopathy or limb girdle myopathy or muscular dystrophy, and without an obvious diagnosis (myotonic dystrophy, dermatomyositis, oculopharyngeal muscular dystrophy, FSH dystrophy) were tested on the initial visit. Those with positive DBS testing had samples sent to Duke University for genetic testing.

## Results

117 patients have been tested to date (number will increase by November 2012). Of these, 5 patients were found to have significant abnormalities on DBS testing. Genetic results are pending.

## Discussion

This approach was designed to further define the clinical phenotypes of patients with Pompe disease. A second objective was to create a system in an adult setting whereby DBS is used routinely, thereby increasing the ability to identify Pompe patients with previously undiagnosed myopathies. The setup is user friendly, eliminates the need for muscle biopsy in a percentage of patients, and can easily be expanded to include patients referred to other neuromuscular specialists in the hospital system, as well as pulmonologists following patients with undiagnosed neuromuscular respiratory failure. Future plans include expanding to include these other patient populations.

